# Variability of assister availability in health insurance marketplace in the U.S.

**DOI:** 10.1186/s12913-018-3285-5

**Published:** 2018-06-19

**Authors:** Jayoung Han, Dongwoo Ko

**Affiliations:** 1 Pharmacy Practice, School of Pharmacy and Health Sciences, Fairleigh Dickinson University, 230 Park Avenue, M-SP1-01, Florham Park, NJ 07932 USA; 2 Marketing, College of Business, Hankuk University of Foreign Studies, Seoul, South Korea

**Keywords:** Affordable care act, Assister, Health insurance, Marketplace

## Abstract

**Background:**

The Affordable Care Act (ACA) established the health insurance marketplaces to provide people the opportunity to obtain healthcare coverage. Assisters have worked with people who may have difficulty understanding the new system and selecting the right plan. This study aims to describe the local availability of assister programs, and examine the factors influencing assister provision.

**Methods:**

The 2016 Small Area Health Insurance Estimates data and a database of assister programs constructed using healthcare.gov were analyzed at the county level. Bivariate analysis by assister provision was performed to determine the differences between the two groups, and the hierarchical generalized linear model was used to examine the factors predicting assister availability.

**Results:**

The study analyzed 2260 counties nested within 35 states. Assister availability largely varied across counties and states. About half of the counties did not provide assisters at all, and the assister provision rate at state level ranged between 19 - 100%. Counties in metropolitan areas were more likely to provide assister programs than rural areas, and so were counties with higher adult uninsured rate or higher uninsured rate among the people with incomes between 138 - 400% of federal poverty level (FPL).

**Conclusions:**

Despite the important role of in-person assistance in plan enrollment, no previous study has examined the local variability of assister program. Our study found a large geographical variation in assister availability, raising concerns about the disparity in access to assister service.

## Background

The Affordable Care Act (ACA) of the U.S. was signed into the law in 2010 to expand the accessibility to health insurance. The law implemented two important policies to reduce the uninsured rate – expanding Medicaid eligibility and establishing health insurance marketplace (hereafter, marketplace). Medicaid has provided low-income population with health insurance for decades. Median eligibility cutoff prior to the ACA was 61% of federal poverty level (FPL) [1] and the ACA expanded the eligibility up to 138% of FPL. States have an option to adopt or refuse the expansion – as of 2018, 33 states have adopted [2].

Marketplace is a newly established individual market in which Americans can purchase health insurance regardless of their preexisting conditions or employment status. People with low- and moderate-income, defined as household less than 400% of the FPL, can receive income-based federal subsidies for coverage. As of March 2016, approximately 11.1 million consumers had obtained health coverage through marketplace and 85% of those received premium subsidies [3].

Although the ACA has decreased the uninsured rate, increased access to care, and improved health outcomes [4, 5], there are still individuals who remain uninsured [6]. Individuals appeared to have psychological (anxiety of selecting a wrong plan, lack of knowledge), and economic barriers (affordability) to enrolling in marketplace plans [7]. Also, some uninsured individuals appeared to be little aware of coverage options and financial assistance, thereby perceiving coverage as expensive [6]. Further, even among those provided information, the uninsured often made enrollment decisions based on incorrect information [6], which underscores the importance of valid information sources. Moreover, the residents of federal marketplace states need to participate in marketplace through the website healthcare.gov that may pose another barrier to enrolling for less literate people.

To help people navigate the health insurance plans, local in-person assistance is available in the health insurance market, which was found to be one of the strongest predictors of plan enrollment [8]. Navigators offer year-round assistance with the enrollment process and provide outreach programs to raise awareness about the marketplace. Navigators receive federal grants and federal training. Certified Application Counselors (CACs) perform the same functions as navigators but are certified through marketplace-designated organizations such as hospitals or community health centers. Enrollment Assistance Program (EAP) enters a contract with Centers for Medicare & Medicaid Services (CMS) to temporarily supplement navigators and CACs during the open enrollment period in the communities with high uninsured rate [9, 10]. Insurance agents or brokers can also provide consumers in-person assistance to the extent states permit and are paid by insurance companies. The website healthcare.gov provides search for assister programs (navigators, CACs, EAP) and agents/brokers separately. In 2016, the majority of assister programs were CACs [10].

About 5000 assister programs during the open enrollment period in 2016 helped about 5.3 million individuals, but the vast majority of assister programs rarely provide statewide services [10], raising concerns over the availability of in-person assistance. Thus, this study described a small area variation of assister availability and examined the factors associated with assister provision at county level.

## Methods

### Data

The study constructed a database for the assister availability using the website healthcare.gov. The study also analyzed data from the 2015 Small Area Health Insurance Estimates (SAHIE), which were released by the U.S. Census Bureau in August 2016.

Assister data were constructed using the healthcare.gov platform that helped in the search for assisters by means of zip code and state [11]. The assisters within 5 miles (narrowest search available) from the entered zip code were first identified, and then the total number of assisters per zip code was entered in an Excel spreadsheet. The zip code level assister was collapsed to create the number of assisters per county. All data were merged by the county federal information processing standard (FIPS) codes. All counties were categorized into metropolitan statistical area (MSA), non-MSA, or rural areas using the delineation files from the census bureau [12].

The SAHIE data provide single-year estimates of health insurance status for all counties in the U.S. The data provide the number of non-elderly individuals in poverty with five income-to-poverty ratio (IPR) categories (at or below 200, 250, 138, 400, and 138% to 400% of poverty) at county level [13].

### Statistical analysis

We created a binary variable of assister provision at the county level as a dependent variable of the hierarchical generalized linear model (HGLM) (counties offering at least one assister programs were coded as 1 and counties offering no assister programs were coded as 0). The proportion of the uninsured among the people aged between 18 and 64 (adult uninsured rate), the proportion of the uninsured among the people with below or at 138% of FPL, those with incomes 138 –400% of FPL, those with incomes above 400% of FPL, and the proportion of the uninsured among females were reported.

We also created a state-level binary variable of Medicaid expansion (counties in Medicaid expanding states were coded as 1 and counties in non-Medicaid expanding states were coded as 0). Counties in Utah, Wyoming, South Dakota, Nebraska, Kansas, Oklahoma, Texas, Missouri, Wisconsin, Tennessee, Mississippi, Alabama, Georgia, Florida, North Carolina, South Carolina, Virginia, and Maine were Medicaid expanding states and the rest were coded as non-expanding states.

The study modeled the county-level assister provision (level 1) nested within state (level 2) using the HGLM to account for the hierarchical nature of the data and the non-normal distribution of the outcome variable. The analysis was performed using PROC GLIMMIX with the binary distribution and logit link. The model fit was assessed by examining the change in the − 2 log-likelihood (−2LL) between models using the Wald test.

## Results

Our sample included 2260 counties nested within 35 states (AK, AL, AZ, DE, FL, GA, HI, IA, IL, IN, KS, LA, MD, ME, MO, MS, MT, NC, ND, NE, NH, NJ, NV, OH, OK, PA, SC, SD, TN, TX, UT, VA, WI, WV, WY). Half of counties were located in the South region and four in ten counties included rural areas. The majority of counties were within states that run federally facilitated marketplace (FFM) (88.2%) and did not adopt Medicaid expansion (67.9%). About half of the counties did not provide any assisters. The most striking difference between counties providing assisters and those not providing them was rurality. About half of the counties providing assisters were located in metropolitan areas (46.9%), while a similar proportion of counties without assisters was located in rural areas (55.1%) (Table 1).

**Table 1:** County characteristics (*n* = 2260)

Variable	All		Assister provided (*n* = 1210)		Assister not provided (*n* = 1051)		*P*-value
			n	%	n	%	
Medicaid expansion							0.027
Yes	726	32.1	413	34.1	313	29.8	
No	1535	67.9	797	65.9	738	70.2	
Rurality							<.001
Metropolitan area	855	37.8	568	46.9	287	27.3	
Micropolitan area	462	20.4	277	22.9	185	17.6	
Rural area	944	41.8	365	30.2	579	55.1	
Region							<.001
West	153	6.8	53	4.4	100	9.5	
Midwest	820	36.3	424	35.0	396	37.7	
South	1173	51.9	648	53.6	525	50	
Northeast	114	5.0	85	7.0	29	2.8	
Marketplace type							<.001
State- partnership	248	11.0	162	13.4	86	8.2	
Federally- facilitated	1993	88.2	1042	86.1	951	90.6	
State based	19	0.8	6	0.5	13	1.2	

**Fig. 1:**
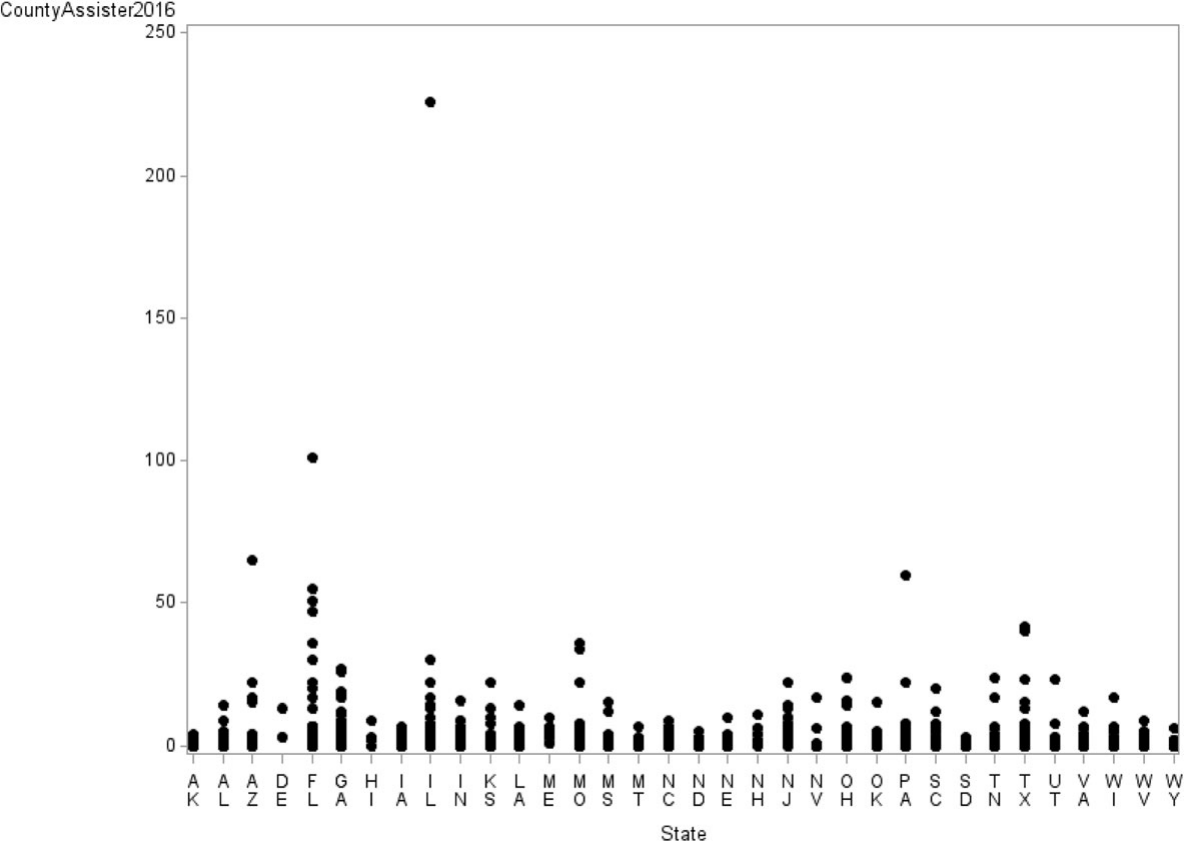
Distribution of the number of assisters by state

The average adult uninsured rate at the county level was 18.3%, which did not differ significantly between counties providing assisters and those not providing them (18.2% versus 18.5%; *p* = 0.330). However, both groups were different in terms of uninsured rate among people with incomes below 138% of FPL (24.1% versus 25.8%; *p* < 0.001) or with incomes 138 –400% of FPL (15.4% versus 15.9%, *p* = 0.035). The number of assisters varied widely across counties and states ranging from 0 to 226 (Fig. 1). The state of North Dakota had the lowest proportion of counties offering assisters (19%). Relatively smaller sized states (DE, ME, NH, NJ) had a high percentage (greater than 90%) of counties offering assisters. Although Medicaid expansion decision has been known to be highly political [14], it does not appear to be associated with assister availability (Appendix).

Table 2 presents estimates for HGLM of the assister provision. Model 1 represents the unconditional model with no predictors and model 2 represents the model with county-level variables. Model 3 includes all variables including state-level ones and interaction terms. The best fitting model (model 3) indicates that counties were more likely to provide assisters in metropolitan areas than in rural areas (b = 0.75; *p* < 0.001), and counties with higher proportion of uninsured adults were more likely to provide assisters (b = 0.16; *p* = 0.013). In addition, counties with higher proportion of uninsured among the people with income 138 –400% of FPL were more likely to provide assisters (b = 0.48; p < 0.001), but those with higher proportion of uninsured among the people with income either below 138% of FPL (b = − 0.25; *p* < 0.001) or above 400% of FPL (b = − 0.75; *p* < 0.001) were less likely to offer assisters. Medicaid expansion status was not associated with the likelihood of providing assisters in general, but counties in Medicaid expanding states that had higher uninsured rate among the people with income 138 –400% of FPL were more likely to provide assisters (b = 0.39; *p* = 0.012).

**Table 2:** Estimates for two-level generalized linear models of Marketplace enrollment

	Model 1		Model 2		Model 3^a^	
	β	S.E.	β	S.E.	β	S.E.
Fixed effects						
Intercept	0.23	0.17	0.68^*^	0.39	0.79	0.50
County Rurality						
Metropolitan			0.71^***^	0.13	0.75^***^	0.15
Micropolitan			0.34^**^	0.13	0.31^*^	0.16
Rural (reference)			–	–	–	–
% uninsured among adults			0.17^***^	0.06	0.16^**^	0.06
% uninsured among people with below or at 138% FPL			−0.25^***^	0.03	−0.25^***^	0.03
% uninsured among people with 138–400% FPL			0.52^***^	0.06	0.48^**^	0.07
% uninsured among people with 400% FPL or higher			−0.75^***^	0.09	−0.62^***^	0.10
% uninsured among female			−0.11	0.08	−0.11	0.09
Medicaid expansion					−0.08	0.80
Medicaid ^*^ 138% FPL					−0.09	0.06
Medicaid ^*^ 138–400% FPL					0.39^**^	0.16
Medicaid ^*^ 400% FPL					−0.73^***^	0.22
Medicaid ^*^ metropolitan					−0.25	0.28
Medicaid ^*^ micropolitan					0.05	0.29
-2LL	2899.71		2646.81		2629.37	
Pearson Chi-square/DF	0.97		0.99		0.99	
Level 2 intercept (covariance parameter)	0.87^***^	0.28	0.55^***^	0.18	0.53^***^	0.18
ICC	0.21		0.14^***^		0.14^***^	

Note. ^*^: *p*<0.01; ^**^:*p*<0.05; ^***^:*p*<0.001= likelihood ratio test significant; ICC = 0.21; values based on SAS PROC GLIMMIX. References of rurality and Medicaid expansion are rural area and no expansion, respectively. % potential enrollees, % uninsured, and % female are the share of non-elderly individuals with 138 to 400% FPL, uninsured, female among the non-elderly individuals, respectively

^a^Best fitting model

## Discussion

To our best knowledge, this is the first study to describe a small area variation in the availability of assisters. Our findings suggest that county-level assister availability was limited and varied largely across counties and states. We also found that counties of Medicaid expanding states with higher uninsured rate among the people with incomes 138 –400% of FPL were more likely to provide assisters.

The findings of our study, which compared counties providing assisters with those not doing so and HGLM results, suggest that a large geographical variation in assister availability may stem from demographic characteristics of each county. More than half of the counties without assisters were located in rural areas while only three in ten counties with assisters were in rural areas. In addition, a slightly higher proportion of counties providing assisters were located in Medicaid expanding states than counties without assisters. Furthermore, the strongest predictor of the likelihood of providing assisters was being located in a metropolitan area.

Assister programs often utilize the existing community organization instead of establishing a brand-new program. Counties including metropolitan areas may have better environment to operate assisters. In addition, assister programs often serve targeted populations and rarely coordinate with each other. Eight in ten assister programs serve specific areas within states, largely targeting people with limited English proficiency or low-income [10]. This is consistent with our results that show a higher likelihood of assisters being available in counties with higher uninsured rate among low- and middle-income residents (138 –400% of FPL).

Our HGLM results also showed that counties with higher uninsured rate among the people with incomes below 138%, who are eligible for Medicaid, and incomes above 400%, which are considered part of high-income populations, were less likely to provide assisters. This is logical as the state Medicaid office would help those eligible for Medicaid with their enrollment process. The high-income population, on the other hand, is likely to obtain health insurance through employer. The possibility of being covered through employer-sponsored insurance changes as income level changes – only about half of people with 100 to 249% of FPL as opposed to 71% of people with 250 to 399% of FPL have employer-sponsored insurance [15].

Medicaid expansion was not significantly associated with the likelihood of assisters being available, but when we take into account the poverty level, it became significant. Medicaid expanding states may be more conscious about the need for assisters in marketplace enrollment while they expand the resources for Medicaid office to handle potentially increased Medicaid enrollment. Alternatively, Medicaid expanding states might have integrated the Medicaid system with marketplace as a part of Medicaid-marketplace coordination.

Access to assisters is important particularly for the uninsured who have little experience with health insurance. The uninsured tend to have little understanding of health insurance terminology [16], which suggests that they may have difficulty navigating the plan that meets their needs best on their own. In addition, the uninsured households tend to spend more on basic needs such as housing or food compared to insured household [17], suggesting that they may not value health insurance as much.

Assisters can tailor the information to individual level, help people understand how health insurance works, and make them understand the value of health insurance, leading to increase insurance enrollment. Assisters were found to increase Medicaid/marketplace application completion rates among the low-income, non-elderly adults in three states [8]. Furthermore, states with higher navigator grants, which is indicative of more rigorous outreach efforts, had a higher marketplace enrollment rate [18].

Moreover, several studies have shown that people remain uninsured because they are not well-versed with financial assistance or eligibility, thereby perceiving marketplace plans as expensive [6, 8]. The application process through the marketplace website may be challenging to people with limited literacy. Assisters could provide people with tailored services and improved awareness of financial assistance that results in increasing marketplace enrollment.

### Limitations

This study did not differentiate between types of assisters (navigators, CACs, EAP) due to a lack of such data. Considering that CACs provide temporary assistance during the open enrollment period, the local availability of assisters may differ at different times of the year.

## Conclusions

The marketplace aims to target non-elderly individuals with low- and moderate-income, who are not eligible for public insurance, and are less likely to have access to employer-sponsored insurance. These people are highly likely to remain uninsured if they do not participate in the marketplace. Assisters help people understand complex insurance information, navigate the plan that meets a person’s needs, and check the eligibility of financial assistance. Our study uncovered a large geographical variation in assister availability, raising concerns about the disparity in access to this critical service, particularly in rural counties. Policy-makers should consider expanding assister programs to promote marketplace enrollment, leading to a reduction in the overall uninsured rate.

## Appendix

**Table 3:** Assister availability by state. Data reports the county numbers analyzed per state and proportion of counties offering assisters

State	Total counties	Number of counties analyzed			Percent counties providing assister	Party affiliation of the Governor
		Total	Assister provided	Assister not provided		
Alabama	67	67	34	33	50.7%	Republic
Alaska	29	15	4	11	26.7%	Independent
Arizona	15	15	9	6	60.0%	Republic
Delaware	3	3	3	0	100.0%	Democratic
Florida	67	67	48	19	71.6%	Republic
Georgia	159	158	120	38	75.9%	Republic
Hawaii	5	4	3	1	75.0%	Democratic
Illinois	102	97	81	16	83.5%	Republic
Indiana	92	92	52	40	56.5%	Republic
Iowa	99	88	32	56	36.4%	Republic
Kansas	105	94	37	57	39.4%	Republic
Louisiana	64	64	31	33	48.4%	Democratic
Maine	16	16	16	0	100.0%	Republic
Maryland	24	1	0	1	0.0%	Republic
Mississippi	82	81	39	42	48.1%	Republic
Missouri	115	115	80	35	69.6%	Democratic
Montana	56	54	15	39	27.8%	Democratic
Nebraska	93	86	18	68	20.9%	Republic
Nevada	17	14	3	11	21.4%	Republic
New Hampshire	10	10	9	1	90.0%	Democratic
New Jersey	21	21	19	2	90.5%	Republic
North Carolina	100	100	59	41	59.0%	Republic
North Dakota	53	42	8	34	19.0%	Republic
Ohio	88	88	66	22	75.0%	Republic
Oklahoma	77	77	38	39	49.4%	Republic
Pennsylvania	67	67	41	26	61.2%	Democratic
South Carolina	46	46	40	6	87.0%	Republic
South Dakota	66	47	14	33	29.8%	Republic
Tennessee	95	95	52	43	54.7%	Republic
Texas	254	234	98	136	41.9%	Republic
Utah	29	28	10	18	35.7%	Republic
Virginia	133	130	49	81	37.7%	Democratic
West Virginia	55	50	37	13	74.0%	Democratic
Wisconsin	72	71	36	35	50.7%	Republic
Wyoming	23	23	9	14	39.1%	Republic

Note. Difference between a total number of counties and a total number of counties analyzed stems from the Small Area Health Insurance Estimates data in which counties with the population less than 50,000 were excluded

## Abbreviations

ACA: Affordable care act
CACs: Certified application counselors
CMS: Center for medicare & medicaid services
EAP: Enrollment Assistance Program
FFM: Federally facilitated marketplace
FIPS: Federal information processing standard
FPL: Federal poverty level
HGLM: Hierarchical generalized linear model
IPR: Income-to-Poverty Ratio
MSA: Metropolitan statistical area
SAHIE: Small area health insurance estimates
